# Remote sociophonetic data collection: Vowels and nasalization over video conferencing appsa)

**DOI:** 10.1121/10.0003529

**Published:** 2021-02-18

**Authors:** Valerie Freeman, Paul De Decker

**Affiliations:** 1 Department of Communication Sciences and Disorders, Oklahoma State University, Stillwater, Oklahoma 74078, USA; 2 Department of Linguistics, Memorial University of Newfoundland, St. John's, Newfoundland and Labrador, A1A 3XB, Canada

## Abstract

When the COVID-19 pandemic halted in-person data collection, many linguists adopted new online technologies to replace traditional methods, including video conferencing applications (apps) like Zoom (Zoom Video Communications, San Jose, CA), which allow live interaction with remote participants. This study evaluated the suitability of video calls for the phonetic analysis of vowel configurations, mergers, and nasalization by comparing simultaneous recordings from three popular video conferencing apps (Zoom; Microsoft Skype, Redmond, WA; Microsoft Teams, Redmond, WA) to those taken from professional equipment (H4n field recorder) and an offline iPad (Apple, Cupertino, CA) identical to those running the apps. All three apps conveyed vowel arrangements and nasalization patterns relatively faithfully, but absolute measurements varied, particularly for the female speaker and in the 750–1500 Hz range, which affected the locations (F1 × F2) of low and back vowels and reduced nasalization measurements (A1-P0) for the female's prenasal vowels. Based on these results, we assess the validity of remote recording using these apps and offer recommendations for the best practices for collecting high fidelity acoustic phonetic data from a distance.

## I. INTRODUCTION

When the COVID-19 pandemic curtailed in-person data collection, some linguists and other social scientists turned to popular video conferencing applications (apps) like Zoom (Zoom Video Communications, San Jose, CA) to continue conducting field interviews (Lupton, 2020). Even before the pandemic, some disciplines have explored the use of video conferencing software for research purposes, citing high participant satisfaction and ease of use ratings for Zoom (Archibald *et al.*, 2019). Two trends from recent years may have positive effects on online data collection. The first is a general improvement in the capacity and stability of internet connections: in the decade between 2007 and 2017, the average speed of internet transmission rates increased from 3.67 to 18.75 Mbps (Akamai, 2017). Relatedly, an initiative in Canada expects to set a national standard for internet speed such that “90% of Canadian homes and businesses will have access to broadband speeds of at least 50 Mbps for downloads and 10 Mbps for uploads” by the end of 2021 (Government of Canada, 2020). The second internet trend is the ubiquity, utility, and increased popularity of apps like Microsoft Skype (Redmond, WA), Microsoft Teams (Redmond, WA), and Zoom (Zoom Video Communications, San Jose, CA). As a result, linguistic data collection is likely to continue expanding outside the phonetics laboratory and field interview, necessitating an evaluation of the quality of user-generated and researcher-facilitated online recordings and remote transmissions. In order for acoustic phonetic researchers to capitalize on these options, we must determine the suitability of such apps, as well as wireless transmission, for collecting spectral measurements common to phonetic analysis.

This paper constitutes part of a larger project on remote sociophonetic data collection. Another part compares recordings made from various popular mobile devices to professional equipment. In this study, we present results from a comparison of simultaneous recordings made over three popular video conferencing apps (Zoom, Skype, and Teams), the same model of Apple iPad (Cupertino, CA) used to make the calls, and professional recording equipment. Our focus is on the acoustic properties of vowel space configurations, mergers, and nasalization.

### A. Background

P.D.D. previously conducted a number of studies on the reliability of formant measurements from lossy audio recording formats (Bulgin *et al.*, 2010) as well as from popular consumer audio recorders and recordings posted to YouTube (De Decker and Nycz, 2011). These studies focused on the fidelity of formant measurements across several recording devices and compressed audio compared to lossless recording formats. Of particular importance are their results concerning recordings sent over Skype VoIP (voice over internet protocol) software, which significantly altered F1–F4 for both of the speakers examined. The male speaker showed a “particularly clear Skype-effect” (Bulgin *et al.*, 2010) that resulted in hyper-spacing or an expanded vowel space: high vowels were made higher (lowered F1) and low vowels were made lower (higher F1), back vowels were more backed (lowered F2), and front vowels were more fronted (higher F2). In contrast, the female speaker in their study exhibited a vowel space stretched at the high-front corner and compressed elsewhere.

In the present study, we expanded the scope of these questions to include internet applications designed for real-time audio-visual speech communication. These are potentially useful tools for sociophonetic fieldwork as they enable two types of spoken language data collections. The first collection, which we refer to as user-generated or caller-recorded, can be managed by participants themselves (Androutsopoulos and Staehr, 2018) either while alone or during an interaction with other participants. The second collection is made up of researcher-facilitated recordings that involve researcher-participant interaction like traditional face-to-face interviews (Labov, 1984) conducted and recorded via a wireless network. When recording or transmitting digital audio online, several technical issues are at play, most notably packet loss and compression effects resulting from speech codecs (Mayorga *et al.*, 2003). While a detailed description of these issues is beyond the scope of this paper, we note that when data transfer speeds are compromised, packet loss will destructively alter the sound quality of a digital recording that arrives on the researcher's computer.

### B. Video conferencing apps and wireless transmission

Zoom, Skype, and Teams, three popular video conferencing apps, were selected for our study. Each is freely accessible online for Mac OS (Apple, Cupertino, CA), Windows (Microsoft, Redmond, WA), iOS (Apple, Cupertino, CA), Android (Google, Mountain View, CA), and Linux systems (Linux Kernel Organization, kernel.org) and features a downloadable client app that allows at least one person to record live calls, making them feasible for researchers to use in recording study participants during remote interviews, discussions, or monologues. All three apps digitize incoming voice signals using a speech codec: Zoom (see Integrated Audio^1^) employs one called Opus, and whereas Skype and Teams used SILK at the time of data collection, Microsoft (Redmond, WA) has announced plans to switch to their own codec, Satin (Salazar, 2020). Once speech is encoded by the application, data are transmitted through a network to the receiving computer in discrete packets which are subject to delays in internet traffic. At this stage, the packets of audio data must be decoded in the proper sequence and in fast succession to achieve a functional conversation across devices, although if transmission rates are low, packets can arrive out of sequence, late, or get lost entirely. According to their website, Zoom's VoIP service is “designed for low latency” in packet delivery (Anderson, 2020), a strategy that would facilitate fidelity of the speech signal. Latency is the time (in milliseconds) it takes for a package to reach its destination. The lower the latency, the higher the call quality, although Zoom states that “latency of 150 ms or less is recommended” (Anderson, 2020). Zoom also “carefully smooth(s) over lost packets when network conditions deteriorate… (and) continues to perform well in environments with up to 45% packet loss… (though) typically, a packet loss of 2% or less is recommended” (Anderson, 2020). In contrast, Microsoft, which owns Skype and Teams, suggests that, among other things, a packet loss rate of 10% or higher is considered poor quality (Arbuthnot, 2018; Rasmussen, 2013). Microsoft also recommends a wireless transfer speed of 1.2 Mbps upload/download for high-definition video to ensure low latency of packet transmission (see Skype Support^2^). Crucially, all three apps respond to network traffic (e.g., changes in bandwidth) in order to maintain a decent user “quality of experience” (Mok *et al.*, 2011), and one study has found that Zoom does so with a “more consistent bitrate” than Teams does (Clopper *et al.*, 2020).

### C. Research questions

To assess the potential for using these apps and wireless transmissions as data collection tools, we must characterize how they affect the fidelity of the acoustic phonetic data that they record. We did so guided by the following research questions:

- (Q1) Apps: Are recordings made by *callers* on popular video conferencing apps suitable for vowel formant and harmonic spectrum (e.g., nasality) measurements? How faithfully do the apps capture these acoustic properties relative to the devices running the apps?
- (Q2) Transmission: Are live recordings collected by video conference call *recipients* suitable for vowel and nasality measurements? How faithfully are these measurements transmitted across internet connections of different strengths?

To answer these questions, we first examined the locations and relative arrangements of vowels in each speaker's F1 × F2 vowel space, plotted in raw Hz to provide direct comparisons across conditions. Second, we inspected patterns and amounts of overlap between pairs of pre-lateral vowels which may be involved in a merger. Third, we compared patterns of nasalization in oral and prenasal /æ/ vowels by plotting the spectral tilt (A1-P0) across the vowel duration.

## II. METHODS

### A. Participants and materials

Due to constraints on allowing research participants onto campus during the initial weeks of the COVID-19 pandemic, only two speakers affiliated with V.F.'s laboratory were recorded: one female in her early 20s from Oklahoma and one male in his early 40s who grew up in Idaho and Arizona. Each gave written consent and was compensated $5 for their 15-minute session. All procedures were approved by the Oklahoma State University institutional review board. Speakers read a word list from which 75 words were extracted for the present analysis, including 3 words for each monophthong before a coronal obstruent, 3–6 words with each non-low vowel before /l/, and 4 words containing /æ/ before /n/.

### B. Recording procedure

Each speaker was recorded on five devices simultaneously (Byrne and Foulkes, 2007), where each device was placed approximately 30–40 cm from the speaker's mouth while the speaker was seated in a sound-attenuated booth on the Oklahoma State University campus. One device was an H4n Pro field recorder to provide “gold standard” recordings (16-bit, 44.1 kHz WAV files) as baselines for comparison. To address Q1 (apps), the other four devices were identical iPad Airs (iOS 12.4.7) with one recording in the iPad's pre-packaged Voice Memos app (32-bit, 44.1 kHz .m4a files) and the other three recording live calls through popular video call apps Zoom (version 5.0.4, 32-bit, 32 kHz m4a audio files), Skype (version 8.49.49, 32-bit, 16 kHz mp4 files), or Teams (version 2.0.11 with the same file outputs as Skype). Skype and Teams only allow one person to record a call (in this case, the speakers' iPads in the booth), but Zoom allows multiple people to record, therefore, the Zoom call was also recorded live by two receivers using laptops in Newfoundland (NL) and New Brunswick (NB) homes (Macbook Airs, OS 10.15). This transmission served as our high-quality internet condition to address Q2 (transmission). A medium-quality condition was achieved by rerouting the H4n WAV recordings using the virtual microphone software Loopback (Rogue Amoeba, 2020). The audio was transmitted from Praat through Zoom and Skype (separately) on a Macbook Air in NB through a wireless internet connection to another Macbook Air on a wireless home network in Oklahoma where the receiver recorded the calls in each app. Finally, a weak Wi-Fi transmission condition was created by similarly sending the H4n recordings over a wireless router in the NB home from a Macbook Pro to a Macbook Air (both running OS 10.15). The receiving laptop recorded the transmissions within Zoom and Skype and was placed far away from the router to substantially degrade the Wi-Fi signal. During these tests, the Wi-Fi signal strength of each receiving computer was logged continuously using an OS terminal command that recorded the RSSI (received signal strength indication) and noise (any interfering signal, e.g., radio frequency interference) values at every other second of the recording duration. These values were used to calculate the signal-to-noise ratio (SNR) of the wireless connection for the receiving computer, which gave us a rough estimate of the transmission success.

### C. Measurements and analyses

#### 1. Processing

Once the word lists were recorded in each app, we converted the audio channels of the recordings to WAV format using the web-based online audio converter (123apps, LLC, 2020) to facilitate subsequent measurements in Praat (Boersma and Weenink, 2020). This procedure re-encoded each audio file using a sampling rate of 44.1 kHz and 16 bit depth (which of course does not improve the audio quality of the apps' original 32 kHz or 16 kHz files nor remove any effects of mp4 or m4a compression). These WAVs were then processed with a plain text transcript through the semi-automated function of DARLA (Reddy and Stanford, 2015), which creates a Praat TextGrid with word and phone boundaries aligned to the audio. Resulting vowel boundaries were hand-corrected before measurements were taken.

#### 2. Vowel formants

Before starting our formant measurements, we visually confirmed all vowel boundaries marked by DARLA and inspected Praat's linear predictive coding (LPC) formant trace for accuracy. As well, the number of formants was adjusted to fit the speaker: five for the male and four for the female with adjustments per vowel as necessary (i.e., typically increasing the number of formants by one for some high-back pre-laterals, totaling about 10% of the male's measurements and about 25% of the female's measurement). A Praat script measured each F1 and F2 at the temporal midpoint for plain vowels and to minimize coarticulatory effects of the surrounding consonants, measured each F1 and F2 at 35% of vowel duration for pre-laterals. The formant range was set to 0–5000 Hz with a window length of 25 ms and dynamic range of 30 dB, and for about half the male's vowels, the maximum formant was increased to 5500 Hz to improve tracking.

Formants from each recording were plotted separately with ellipses of 1 standard deviation (SD) around vowel means using NORM (Thomas and Kendall, 2007). Obvious outliers far from the ellipses were checked in Praat for measurement errors and corrected. We did not normalize formant values because our analyses were speaker-internal. We then replotted the formants using the phonR package (McCloy, 2016) in *R* (*R* Core Team, 2020). Separate plots were made to address each research question for each speaker and condition. These plots show either the overall vowel space outlined by the pre-coronal monophthong means or potential mergers with ellipses of ±0.5 SD around vowel means.^3^ Plots were inspected visually to compare locations and relative arrangements. Finally, we conducted analyses of variance (ANOVAs) and linear regressions using the lme4 and afex packages (Bates *et al.*, 2015; Singmann *et al.*, 2020) in *R* (*R* Core Team, 2020) to determine whether any app affected formant measurements.

#### 3. Anticipatory nasalization

Our second comparative analysis examined the effects of remote recording apps and wireless transmission on acoustic properties smaller than the broad bandwidths associated with formant frequencies. For this we looked at A1-P0, a spectral tilt measurement of two harmonic amplitudes found in the lower frequency range. This measurement is often examined in studies of anticipatory vowel nasalization in English and inherent vowel nasality in French (e.g., Chen 1995, 1997; Styler and Scarborough, 2018). While A1-P0 is extremely variable across speakers, making a comparison of absolute values impossible, previous studies have shown it is relatively higher in oral contexts and lower in nasal contexts. Anticipatory nasalization can be categorical or gradient (Cohn, 1993). Evidence of a categorical rule would show lower A1-P0 values at the onset of a prenasal vowel compared to relatively high values throughout the duration of a pre-oral vowel. A gradient, phonetic implementation would reveal A1-P0 starting out relatively high and subsequently decreasing over the duration of a prenasal vowel.

In this study, we looked at the anticipatory behavior of A1-P0 over the duration of the low-front lax vowel /æ/. In a number of North American varieties of English, /æ/ exhibits the “short-a split” in which it is typically tensed and raised before nasals and lowered or retracted in oral contexts (Labov *et al.*, 2006). Preliminary work on speakers of Canadian English indicates categorical anticipatory nasalization may be a distinguishing feature of prenasal tensing in the short-a split (De Decker, 2014). To assess the usefulness of video conferencing apps for questions of vowel nasalization, we examined whether each app and transmission condition reliably preserved the A1-P0 patterns produced by our two test speakers.

The A1-P0 measurement procedure was conducted using a Praat script (Styler and Scarborough, 2018) that detects and compares, A1, the amplitude of the harmonic closest in frequency to the first formant and, P0, a low-frequency harmonic referred to as the nasal peak (Chen, 2000). To document the process of anticipatory nasal coarticulation, measurements were taken at ⅓ and ⅔ of each token's vowel duration. Measurements were connected in plots to illustrate the direction of the A1-P0 slope across these two timepoints under each of the recording conditions. A total of seven tokens were examined per speaker per condition, three from the oral context and four from the nasal context.

## III. RESULTS

Before reporting our results, we sketch out the design of our comparative analyses. Both formant (Secs. III A–III C) and nasalization (Sec. III D) analyses consisted of comparisons across the following recording conditions:

1. *App comparison*. We compared recordings created by the caller on each of the video conferencing apps running on an iPad Air. Because this condition used four identical iPads and involved no transmission across a wireless network, results represent a test of each app's speech codec alone. A brief comparison was made with the iPad's default voice recording software to determine if any observed differences were due to the online apps or the standard technology of the iPad.
2. *Strength of wireless transmission.* We compared recordings made by video conferencing call recipients over three different qualities of internet connection:
  (a) *Strong*: Live transmissions from the laboratory in Oklahoma to a laptop computer in NB achieved the best SNR (42 dB, SD = 1), representing a strong network transmission under ideal internet traffic. (The receiver in NL appeared to have a similar network strength and was also treated as a strong connection, although a technical difficulty prevented the signal strength logs from being recorded.)
  (b) *Medium*: Next, the H4n audio files rerouted from the NB network back to an Oklahoma network registered a SNR of 27 dB, SD = 1 on the receiver's laptop.
  (c) *Weak*: The lowest SNR (13 dB, SD = 4) was achieved on the wireless network in NB by placing the receiving laptop at a substantial distance from the wireless router.

Each of these comparisons allowed us to test our overall question: Do recordings collected over video conferencing apps capture phonetic information faithfully enough to use in acoustic research?

### A. Vowel space shape

#### 1. App comparison

**FIG. 1.**
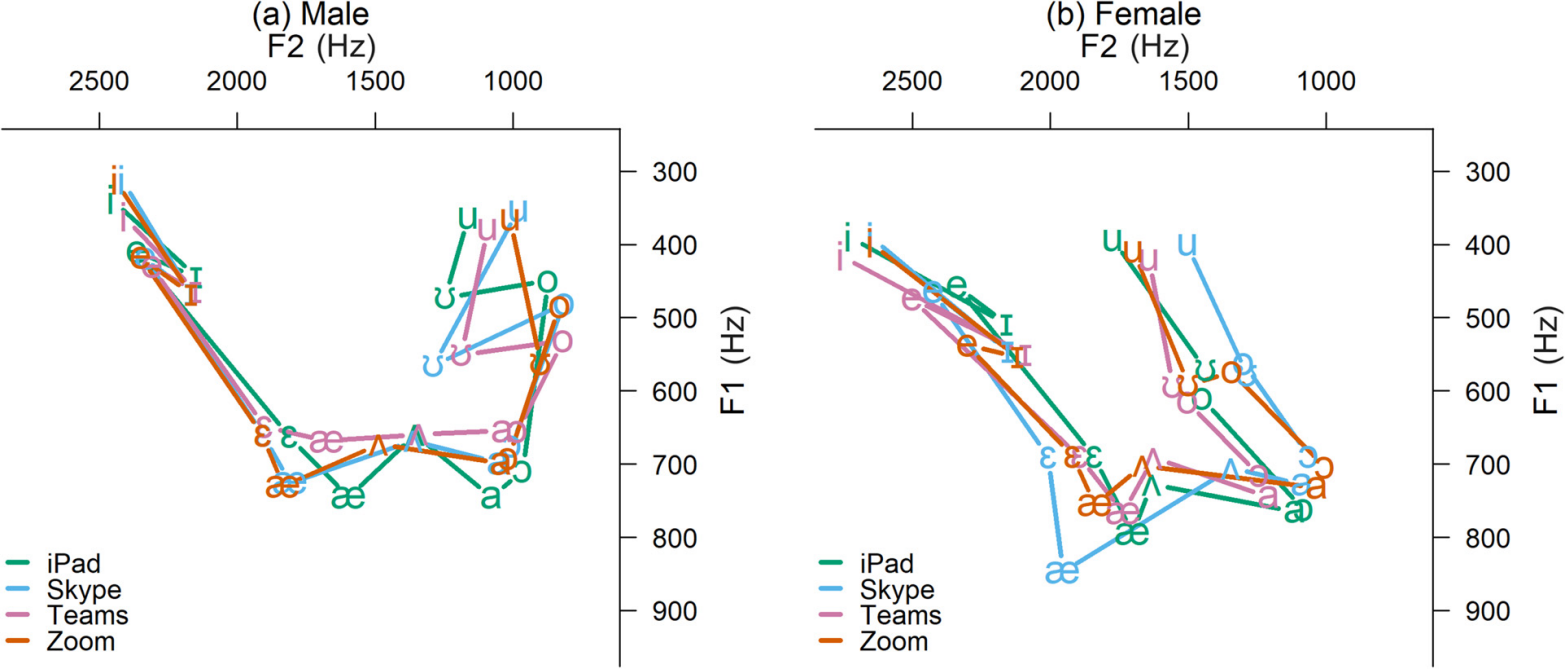
(Color online) Caller-recorded vowel spaces from three apps (Zoom, Skype, Teams) and the same model of iPad that ran the apps.

Figure 1 shows the vowel spaces measured from the four identical iPads with each video conferencing app recording from the caller's end in the booth with each speaker. First, we should note that the offline iPad software recorded slightly smaller vowel spaces than the H4n professional recording equipment recorded. The greatest deviations were in frequencies between about 750 and 1500 Hz, which had the effect of raising the male's low-front /æ/ and high/mid-back /o,ʊ/ and the female's low /æ,a/. Using the iPad recording as a basis for comparison, all three apps maintained each speaker's relative vowel configurations but with some deviation in the absolute formant values, particularly within the range of about 750–1500 Hz, which again affected low and back vowels. Zoom was fairly accurate overall for both speakers, whereas Teams was the most accurate app for the female but the least accurate app for the male (compressing the low and back vowel space), and Skype was the most accurate app for the male but the least accurate app for the female (expanding all but the high-front vowel space).

#### 2. Strong connection

**FIG. 2.**
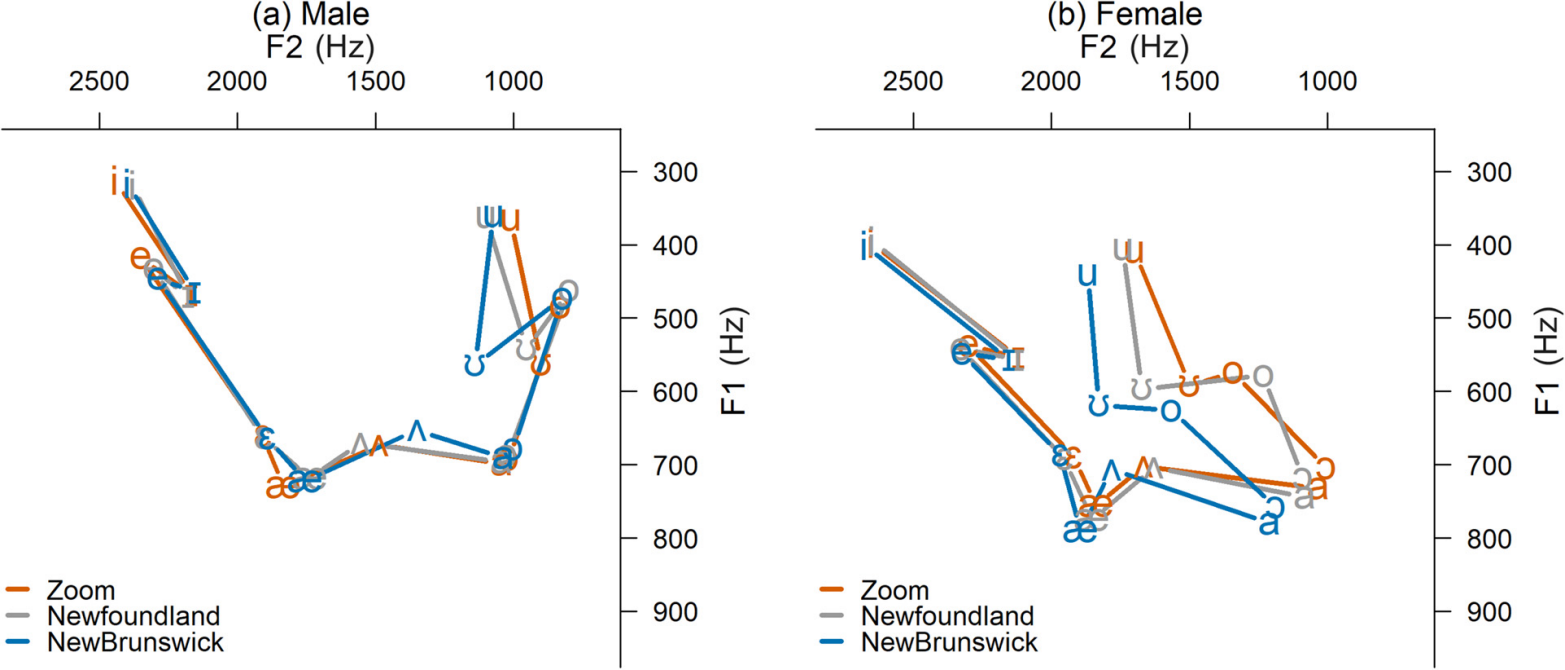
(Color online) Receiver-recorded vowel spaces captured during live Zoom calls over strong internet connections compared to the caller's Zoom recording.

Because Zoom allows both the caller and receivers to record a meeting, remote receivers in NL and NB recorded the Zoom call live over strong internet connections. Figure 2 compares formants from these recordings to those from the Oklahoma caller's Zoom recording (as in Fig. 1), showing that most vowel formant information was faithfully transmitted, especially for the male. However, the female's already fronted back vowels were further fronted in the transfer to the NB receiver.

#### 3. Medium and weak connections

Because Skype and Teams do not allow multiple participants to record at once, each speaker's recording taken on the H4n field recorder was later played as a computer-internal audio source during calls over each app on a medium-SNR internet connection from NB to Oklahoma where the receiver recorded the transmission within each app. All three apps recorded formants from these transmissions extremely faithfully. We also ran a similar test within the NB location over a weak internet connection. Again, both Zoom and Skype recorded formants from these transmissions extremely faithfully regardless of the poor SNR over the weak Wi-Fi signal (technical difficulties prevented the same test with Teams).

### B. Merger: Vowel overlap patterns

**FIG. 3.**
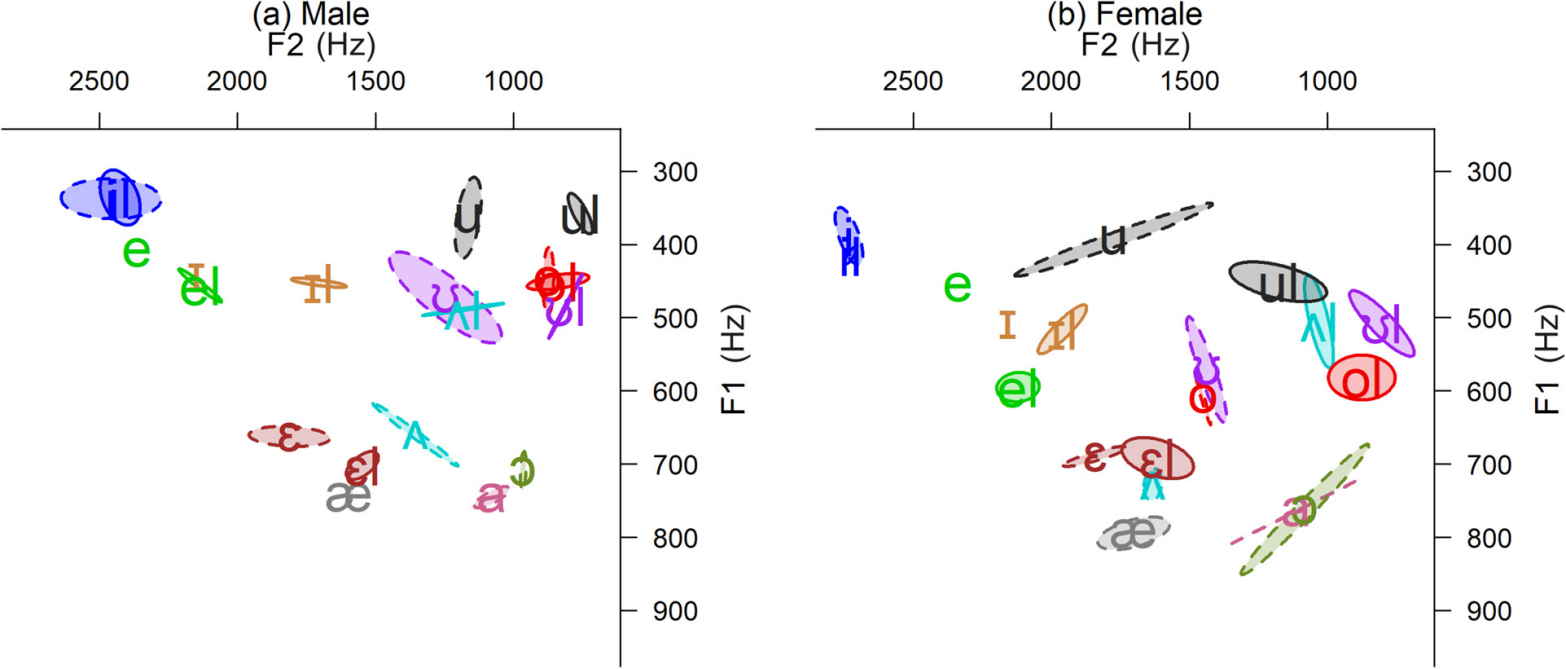
(Color online) Plain and pre-lateral vowels from the offline iPad with 0.5 SD ellipses around means.

Figure 3 shows each speaker's vowel space with non-low pre-laterals separated as recorded from the offline iPad. We again note that the offline iPad (or its software) compressed both speakers' F1 ranges, which particularly affected the arrangements of their low-front /æ/ and mid-back vowels (as well as the male's /el/), so that they appear raised and closer to their higher neighbors than that which was recorded by the H4n field recorder. Even so, each speaker's merger patterns remain clear. Front vowels were slightly backed and lowered before /l/ as was expected due to anticipatory coarticulation, and none were involved in pre-lateral mergers (*feel-fill, sale-sell*). Low-back vowels were close or overlapping, indicative of a *caught-cot* merger, as expected for both speakers' regions (male, West; female, Oklahoma, a mix of Southern and Midland; Bakos, 2013). The female's back rounded plain vowels were fronted, especially /u/, whereas the male had only a slightly fronted /u/ and /ʊ/ but not /o/. For both speakers, /ʊ/ was low, appearing front of /o/. These patterns were faithfully represented by all apps and transmission conditions.

Back rounded vowels before /l/ were *not* fronted and appeared in the far back vowel space at similar heights as their plain counterparts. The male's /ol/ and /ʊl/ were both backed to overlap plain /o/, resulting in a *bull-bowl* merger, but the female's /ʊl/ was backed even farther and higher than her /ol/, suggesting a near-merger. These patterns were captured by all apps and transmissions, but the height of the central pre-lateral /ʌl/ was more variable across conditions. For both speakers, /ʌl/ was shifted up and back toward the mid-back pre-laterals but to different degrees. The male's /ʌl/ was lower and more front than *bull-bowl* /ol-ʊl/, suggesting a shift or intermediate stage toward a three-way merger, but its exact location and distance from *bull-bowl* varied considerably across apps and conditions as did the location of the centralized plain /ʊ/. The female's /ʌl/ was shifted farther, appearing higher and more front than her /ol/, partway toward the lowered /ul/. This arrangement was recorded by Skype, but Zoom, Teams, and both live recipients of Zoom (as well as the professional H4n device) showed /ʌl/ closer to /ol/, suggesting a near-merger of *cull-coal* but not *cull-cool*. (Note that in another test, several other recording devices showed /ʌl/ at an intermediate location similar to that from the iPad and Skype, so it appears that this mid-back area of the vowel space is particularly vulnerable to variations between devices.)

### C. Vowel formant faithfulness

For an overall view of how our app and transmission conditions affected formant measures, we ran three-way ANOVAs and linear regressions for F1 and F2 separately. Three-way ANOVAs showed significant main effects and all interactions for condition, speaker, and vowel (with plain and pre-lateral vowels as separate qualities) for both F1 and F2, indicating that the apps and transmissions differentially affected the formant measures for each speaker (see Table I in the Appendix). Linear regressions with the offline iPad as the reference condition showed that the caller's Skype and Teams recordings accurately reflected both formants, whereas the caller's Zoom recording exhibited distortion in F1 but not in F2 (see Table II in the Appendix). For the live Zoom calls over a strong internet connection, we set the reference condition to the caller's Zoom recording; both F1 and F2 were faithfully transmitted through Zoom from caller to receiver (see Table III in the Appendix). With the H4n recording set as the reference condition, both formants were accurately transmitted from computer-internal audio through Zoom and Skype to receivers with both moderate and weak internet connections (see Tables IV and V in the Appendix), and Teams showed deviation for F1 but not for F2 over the moderate connection (see Table IV in the Appendix).

Finally, we offer a side note on variation in formant tracking accuracy via the number of outliers that we corrected after taking initial formant measurements for each recording. The offline iPad produced 8 outliers for the male and 11 for the female. From the caller's Zoom and Skype clients, we corrected 3–6 vowels for each speaker, but there were 13 (male) and 17 (female) outliers from Teams. The live Zoom receivers each recorded 8–11 outliers per speaker over their strong internet connections. The H4n recordings produced 5 outliers per speaker; when these recordings were sent as computer-internal audio, the receivers over medium-strength internet connections recorded 6–8 outliers from each app for the male but more for the female: 9–10 from Skype and Teams and 13 from Zoom. The recordings from the weak connection were the most accurate with only 2–4 outliers each.

### D. Anticipatory nasalization

**FIG. 4.**
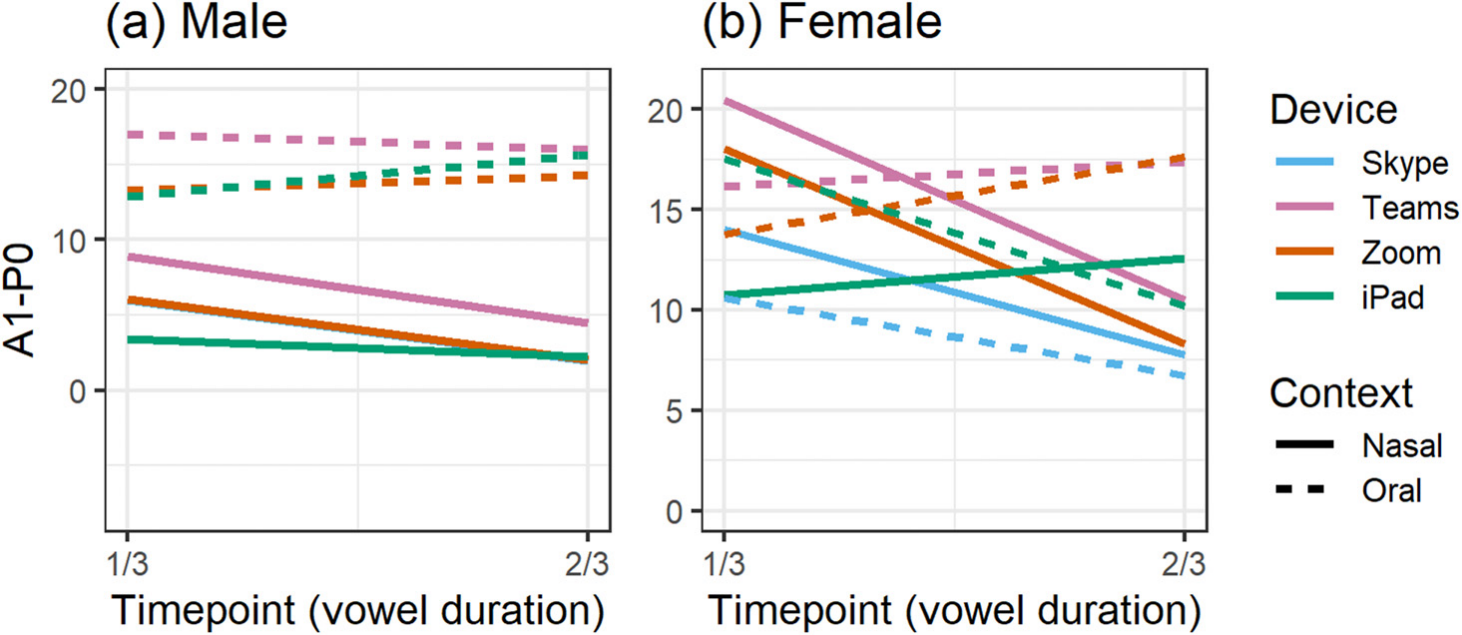
(Color online) Caller-recorded A1-P0 across vowel duration from three apps (Zoom, Skype, Teams) and the same model of iPad that ran the apps.

We turn now to our analysis of vowel nasalization of /æ/ before /n/ compared to /æ/ following oral consonants. Figure 4 illustrates the time-varying patterns of A1-P0 for each of the caller-recorded conditions using Skype, Teams, and Zoom compared to the offline iPad. Measurements taken from the offline iPad's voice recorder software were mostly consistent with other devices we have tested, including a professional Focusrite audio interface powering an Audio Technica 2021 microphone (Audio Technica, Tokyo; but not the H4n used as our control field recorder). The iPad preserved the broad A1-P0 distinction by the context produced by the male speaker [Fig. 4(a)] as well as the converging pattern exhibited by the female speaker [Fig. 4(b)].

#### 1. App comparison

Figure 4(a) also shows comparable patterns across all apps for the male speaker. Whereas each app produced slightly different A1-P0 values, none of them significantly altered the oral-nasal pattern that unfolded over time as recorded by the offline iPad. There was a general rising of A1-P0 approaching the oral stop and the expected falling upon approach to the nasal stop. This suggests that A1-P0 accurately reflected anticipatory nasalization in /æ/. In addition, the A1-P0 differences found at the first measurement point were consistently captured by each app. It is worth noting that the ⅓ timepoint is the furthest point from the nasal source and should be less affected by the following nasal consonant than the ⅔ point. However, contextualized nasalization was present early on in the vowel, reflecting the operation of anticipatory nasalization as a phonological/categorical rule, a pattern identified by Cohn (1993) and consistent with preliminary results found elsewhere (De Decker, 2014).

An overall pattern is less clear for the female speaker [Fig. 4(b)], but the apps seemed to show a distinction by context such that A1-P0 decreased before the nasal /n/. This was interesting and unexpected because it was not the pattern captured by the iPad's offline Voice Memos software. Unlike the male pattern, A1-P0 differences were not realized at the initial timepoint, but rather they were distinguished over the course of the vowel in each of the video conferencing apps. Our two speakers, then, appeared to employ different nasalization strategies in producing /æ/. Whereas the male may have a categorical distinction, the gradual increase/decrease in A1-P0 by context suggests that anticipatory nasalization of /æ/ for the female might operate at the level of phonetic implementation, that is, gradually over the duration of the vowel. It is interesting that the conferencing apps produced the expected pattern of increasing nasalization into a nasal consonant when the iPad's default software did not.

#### 2. Strong connection

**FIG. 5.**
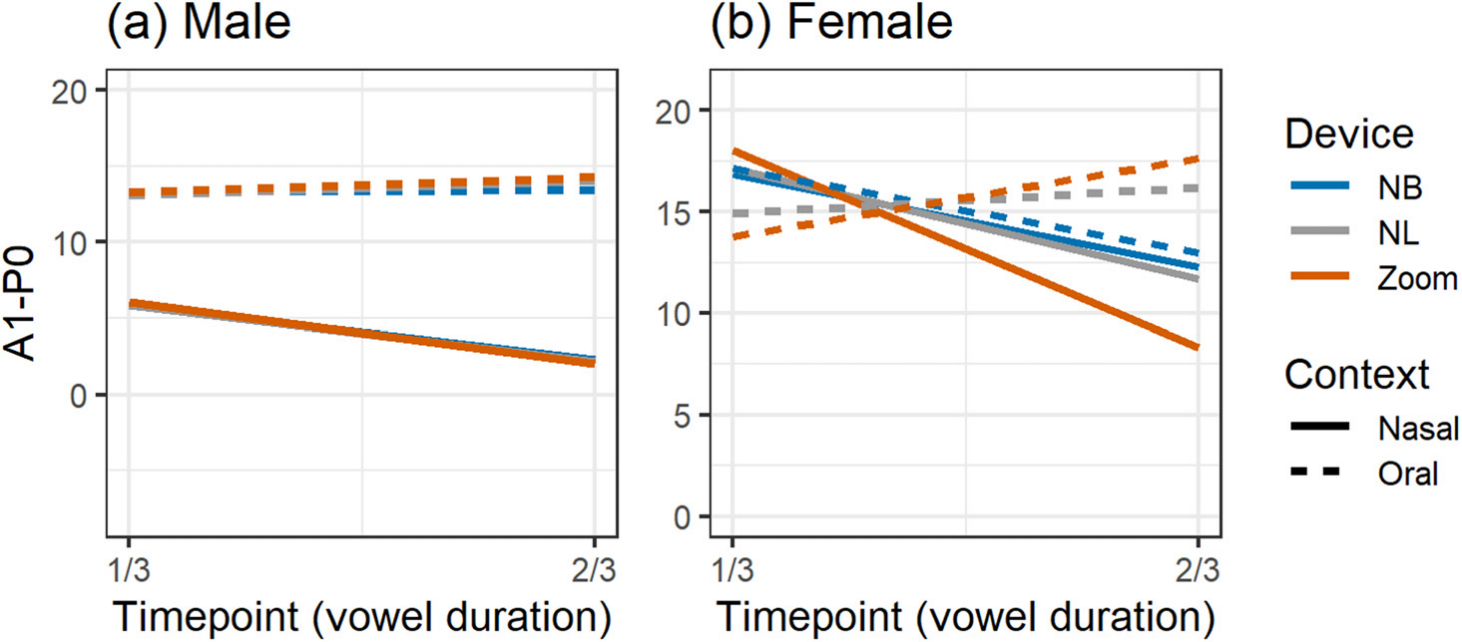
(Color online) Receiver-recorded A1-P0 across vowel duration during live Zoom calls over a strong internet connection compared to the caller's Zoom recording.

Figure 5 compares the effects of a live internet transmission on nasality measurements as recorded live within Zoom by the video call receivers with strong internet connections in NB and NL. Most striking was how the transmissions of nasalization patterns were exact for the male speaker [Fig. 5(a)]. Similar to the finding above, patterns of anticipatory nasalization for the female speaker were inconsistent across the high-quality connection [Fig. 5(b)]. A1-P0 values in the audio received by the laptop in NL generally conformed to the values in the caller's Zoom recording, showing a decrease in the nasal context and an increase in the oral context over time. In contrast, the transmission to NB essentially erased the oral-nasal distinction with both starting high at the ⅓ timepoint and decreasing at ⅔ of vowel duration. In short, Zoom faithfully transmitted our male speaker's nasalization patterns—but not our female's—from caller to receivers across networks with high SNRs.

#### 3. Medium connection

**FIG. 6.**
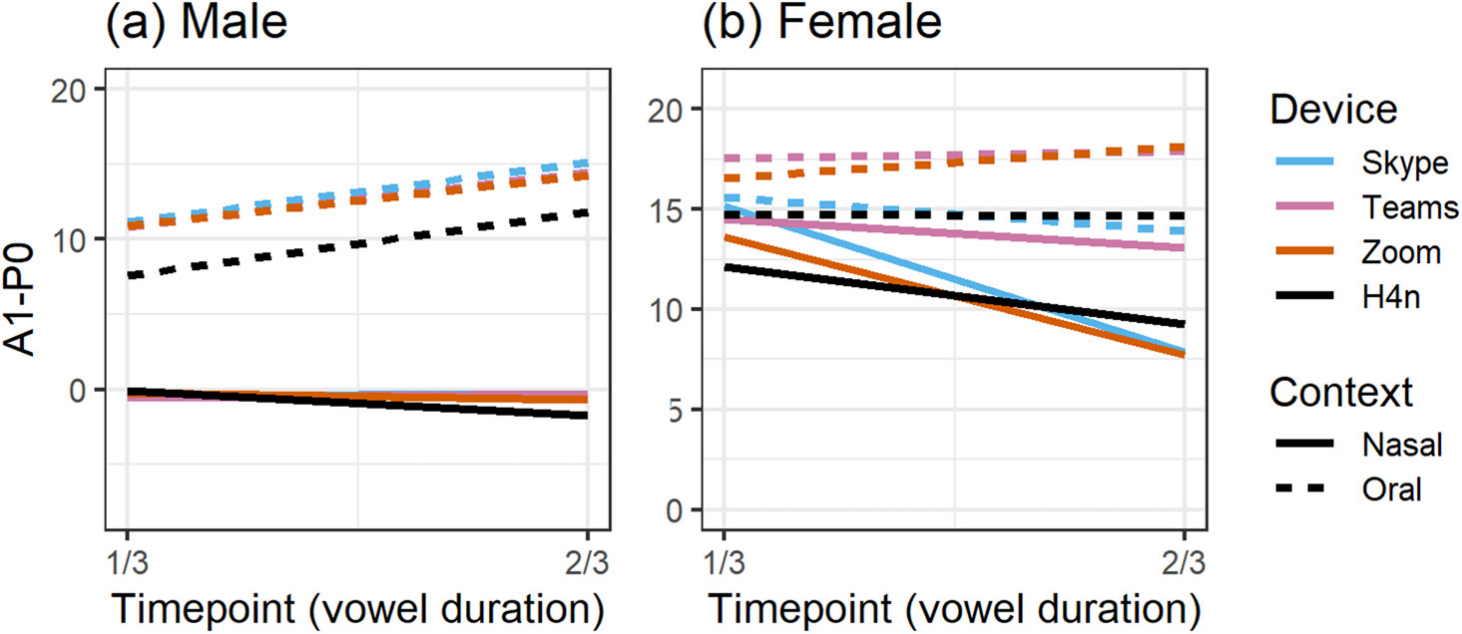
(Color online) Receiver-recorded A1-P0 across vowel duration over a moderate-SNR internet connection as recorded within three apps compared to the H4n recording transmitted through each app.

Figure 6 shows A1-P0 values as received by each app over a medium-quality internet connection compared to the H4n recording that was sent through each app. Under this condition, the SNR for the wireless router receiving the transmission was calculated at 27 dB, SD = 1 (good but approaching low). Patterns found in Fig. 6(a) show faithful reproduction for the male speaker in both oral and nasal contexts across each of the transmissions with one exception: A1-P0 values were slightly increased in vowels preceding oral stops. The slope for the nasal context, however, was nearly identical across all app transmissions. In Fig. 6(b), each of the transmissions broadly replicated the contextual patterns from the H4n source with higher and relatively stable A1-P0 values in the oral context and lower and decreasing values in the nasal context. Importantly, the oral and nasal slopes matched the input, although the Teams app introduced some deviation in the nasal context at the second timepoint. It is interesting that Skype and Teams produced different results despite using the same underlying technology.

#### 4. Weak connection

**FIG. 7.**
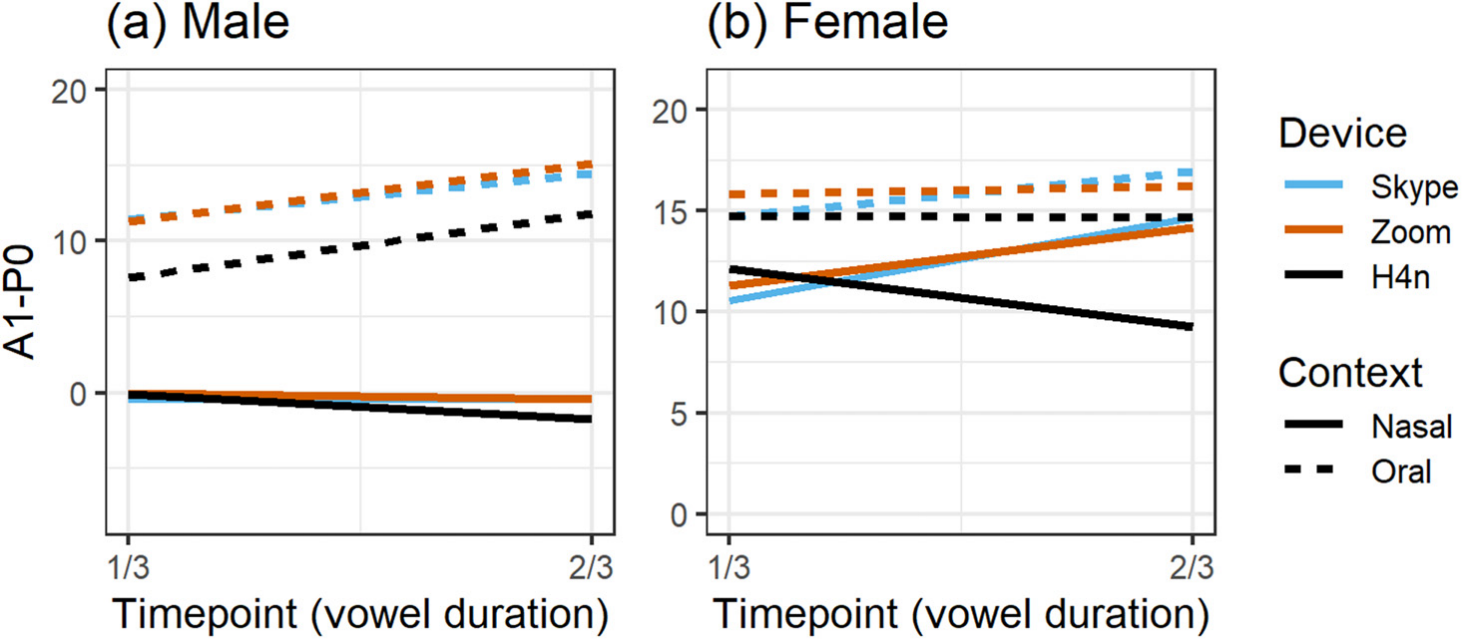
(Color online) Receiver-recorded A1-P0 across vowel duration over a weak internet connection as recorded within two apps compared to the H4n recording transmitted through each app.

Finally, the weak-quality wireless transmissions (SNR = 13 dB, SD = 4) are shown in Fig. 7. Once again, the apps preserved the oral-nasal distinction for the male speaker with a slight but uniform increase in the absolute values across timepoints in the oral context when compared to the H4n source. However, the weak Wi-Fi transmission had a strong effect on the A1-P0 measurements taken from the nasal context in the female recordings, reversing the pattern of anticipatory nasalization found in the H4n recording and the higher-quality transmissions above.

## IV. DISCUSSION

### A. Summary

In this study, we evaluated three popular video conferencing apps (Zoom, Skype, Teams) that could be suitable for remote data collection. Our motivation stemmed from physical distancing practices currently in place which prevent researchers from collecting data using traditional face-to-face methods. We focused on the acoustic properties of two speakers' vowel spaces and a spectral tilt measure of anticipatory nasalization.

From our tests involving vowel spaces, we can cautiously conclude that video conferencing apps record and transmit formant information fairly accurately, making them viable tools for remote phonetic data collection, especially regarding relative arrangements of a speaker's vowel classes or broad categorical determinations of merger (e.g., separate, shifted, near/merged). However, in another part of our project, we found that mobile devices like iPads and smartphones produced recordings with deviant formant frequencies between 750 and 1500 Hz, which affected back and low vowels to a greater extent than the deviations recorded by our apps in the same frequency region. If any alterations are caused by hardware factors, such as microphone quality or internal pre-processing, they will be passed to whatever app a caller uses, thus, using a laptop or external microphone rather than a tablet or smartphone may be more important than the choice of app, especially for research questions that rely on precise vowel locations, distances, or amounts of overlap.

To best make sense of the anticipatory nasalization results, consider the differential treatment of the two speakers analyzed in this study. In terms of reliability, each of the apps did a better job of preserving anticipatory nasalization in the speech of the male speaker. Patterns found in the offline recordings (i.e., iPad or H4n) persisted in each of the app recordings, although with notable differences in the absolute level of A1-P0. Measurements from the female speaker revealed an unexpected change: while the native iPad software introduced an anticipatory nasalization pattern not present in recordings from a control device, the live Zoom transmission from the iPad did not produce that error, rendering the live recordings compatible with those of the control. When we lowered the SNRs of internet connections transmitting prerecorded sound files, the A1-P0 measurements from our male speaker's audio remained remarkably consistent under all three signal strength conditions, but transmitting the female's audio produced recordings that varied considerably by signal strength with greater reliability over the strong and medium-quality connections. These findings suggest that for some voices, Zoom, Skype, and Teams could facilitate a reliable analysis of nasalization where A1-P0 is measured, but they may introduce unexpected alterations with other voices.

### B. Limitations and future work

Before concluding, we point out some methodological issues not addressed so far. First, we have noted a general deleterious effect of slow transmission speed and low bandwidth on packet loss without systematically examining packet loss *per se*. Rather, we took the SNR of the receiving wireless router as a global indicator of the strength of the transmitted signal. Given that all three apps faithfully transmitted computer-internal audio over both strong and weak Wi-Fi connections, it may be that our Wi-Fi connections were not poor enough to show any additional distortions that the apps may apply to save bandwidth. In future work, we will consider other methods of degrading the connection signal, such as through a freely available network emulator package, to test the effects of a greater range of transmissions that may be common during data collection with remote participants. This software will also measure packet loss directly. However, our highly faithful transmission of prerecorded audio files was a pleasant surprise with a secondary benefit. It suggests that some types of perception studies could be conducted over video conferencing apps (as long as precise auditory sensitivity or control is not required for the study)—something else to test in future work.

Second, the language of this paper has referred to variation in reproduction of acoustic measurements across apps and wireless network qualities as distortions, destructions, and alterations, which might imply a certain *agency* to the apps or network. In fact, this variation is likely a combination of digital signal processing, internet connectivity, and Praat's ability to accurately estimate quantitative values for the phonetic features we examined. It should also be noted that these distortions may not have linguistic consequences. For example, the reported decreases in the F1 of a low-back vowel would not necessarily correlate with a perceived change in the identity of that vowel. Similarly, the observed decreases to A1-P0 taken from some of the male speaker's recordings will likely not result in a listener hearing a more nasalized vowel. Whereas the finer perception of speech transmitted using these video conferencing apps remains an avenue for future research, the exact cause of any noted deviation in acoustic measurements should be interpreted as part of a complex system where microphones, speech codecs, network activity, and spectral estimation parameters all play a contributing role in the numerical modelling of speech production.

Third, our data are limited to two speakers of two dialects of American English, which might temper some of the claims we have presented here. Although there is no perfect number of speakers to include in a study like this—as we do not know all of the ways that acoustic measurements might be affected—the inclusion of two speakers with relatively different harmonic structure and resonance properties allowed us to determine that the consequences of using these apps are not necessarily widespread or destructive to the point of unusability. Rather, much of the harmonic and formant frequency ranges examined here remain stable.

Last, we note that we only tested two spectral measures here in a highly controlled laboratory sound booth. It is possible that any combination of voice qualities, equipment, environment, and apps could affect other measures differently. Future work with these or other measures should include more speakers and examine interactions with environmental factors like speaker–device placement and video conferencing apps' handling of background noise. In our focus on simulating everyday speakers' use of free software on personal mobile devices, many technical aspects of hardware, software, and transmission were not examined; future work could manipulate factors like microphone quality, audio compression, codec processing, and packet loss to make recommendations for the selection (and possible modification) of mobile devices, apps, and recording conditions to increase reliability across research participants in remote settings. As the goal of our study was to provide a rapid assessment of the dominant form of data collection during the COVID-19 pandemic, we were primarily concerned with starting the conversation among our colleagues about a range of issues they should consider before collecting and analyzing their own spoken language recordings. With this in mind, we turn to our final contribution: practical recommendations for using video conferencing apps for sociophonetic data collection.

### C. Conclusions and recommendations

Given the reasonably minor variation in formant values introduced by our three test apps, recordings made over video conferencing apps may be suitable for research questions involving relative arrangements of vowels and categorical determinations of merger, but more caution is warranted for questions that rely on small differences to determine distance or amount of overlap, particularly among low and back vowels (e.g., Nycz and Hall-Lew, 2013).

It is less clear if these apps can be trusted in studies of nasalization or what steps can be taken to mitigate app-related distortion. Our male speaker's nasalization patterns were consistent across conditions (with variation in absolute values), but further study is needed to determine the cause of variation in our female speaker's patterns—with only one speaker of each gender, we cannot say if the differences between our speakers are generalizable to others based on gender, age, fundamental frequency, or other factors.

From our results, we make the following recommendations for researchers using video conferencing apps for data collection (with the caveat that these should also be systematically tested for their effectiveness in future work):

(1) Because all three apps performed similarly, any of them could be suitable for spectral measures. If the measure of interest has been shown to be comparable across apps, base the choice on participant familiarity or app availability and friendliness to new users.
(2) If it is unknown how the measure of interest is affected by different apps, consider running a quick test with a few speakers and simultaneous calls on identical devices. If the measure of interest is systematically affected by certain apps, limit future protocols to the most suitable app. If the app of choice affects one type of speaker more than others, consider adjustments that could be calculated to improve comparability across speakers.
(3) Hardware may be a bigger factor than choice of app. Ask participants about the devices they use and include this as a factor in *post hoc* statistical analysis. If participants are likely to be comfortable using a laptop, consider asking them to use that rather than a tablet or phone. However, it is wise to place high importance on participant familiarity with their device and app—much time can be spent in frustration trying to manage an unfamiliar setup.
(4) There are several options for dealing with poor connections over live calls. Ask the participant to record the call on their end so the recording is collected without being sent across the internet connection. (If using Zoom, the researcher can also record the call as a backup.) Consider asking the participant to record the session on a second device (e.g., cell phone) either in addition to recording in the app or instead of recording in the app. This would allow interaction and researcher instruction over the conferencing app while making an uninterrupted, higher-quality recording on an offline device. If video is not necessary between researcher and participant, turn off the cameras to save bandwidth or improve audio recording quality. Researchers can also monitor SNR levels while receiving a call to assess signal strength throughout the transmission and then report the results as a potential influence. There are a number of ways to track network activity; for the convenience of Mac OS X users, we have included our Wi-Fi logging and SNR calculation commands in the Appendix (Code 1 and Code 2). Where possible, this procedure can also be extended to the participant's device to monitor connection quality of the source network.
(5) Finally, we mentioned that perception studies may be feasible over video conferencing apps, for example, by sending stimuli as internal audio from the researcher's computer for participants to hear over a live call. Although this would have the same challenges as any field situation where researchers cannot control participant equipment or environment, our tests strongly suggest that transmitting audio over a live call is comparable to other methods of providing stimuli to remote or asynchronous participants (e.g., embedding audio in online surveys or web pages). Using a conferencing app has the advantage of allowing researcher interaction and real-time observation during the procedure.

COVID-19 has provided the catalyst that we all needed to overcome our discomforts with video calls, both as researchers and everyday users. Zoom has quickly become an everyday tool for work, school, and socializing—and with a critical mass of users who now use it daily—an easy tool for linguistic research whether via observation or controlled elicitation. Video conferencing apps allow live interaction with remote participants and have great potential for reducing the observer's paradox, especially when participants record group interactions without the researcher present. As an example, V.F. is conducting a separate study using participant-only Zoom group discussions in the hopes that speakers feel less inhibited discussing language attitudes without the authoritative (or feared-to-be-prescriptive) presence of an out-group professor. Even with the eventual return of in-person interaction, we argue that linguists should continue to leverage the ubiquity of VoIP technology and video conferencing apps to reach more participants and diversify our methodological toolkits.

## Footnotes

^1^ See “Integrated Audio makes your meetings easy,” available at https://zoom.us/docs/doc/Zoom-Premium-Audio.pdf (Last viewed August 24, 2020).

^2^ See “How much bandwidth does Skype need?,” available at https://support.skype.com/en/faq/FA1417/how-much-bandwidth-does-skype-need (Last viewed August 13, 2020).

^3^ This ellipse size was chosen for clarity of presentation; with so few tokens, larger confidence intervals displayed very large ellipses with great overlap.

## APPENDIX

See Tables I–V.

**TABLE I.** Results of three-way ANOVAs with condition, speaker, and vowel as fixed effects.

F1	df	*F*	*p*
Condition	11	21.90	<0.001
Vowel	18	2179.11	<0.001
Speaker	1	3079.73	<0.001
Condition:vowel	198	4.04	<0.001
Condition:speaker	11	12.61	<0.001
Vowel:speaker	18	35.08	<0.001
Condition:vowel:speaker	198	1.58	<0.001
Residuals (df denominator)	1192		
F2	df	*F*	*p*
Condition	11	10.87	<0.001
Vowel	18	6357.19	<0.001
Speaker	1	2884.08	<0.001
Condition:vowel	198	5.36	<0.001
Condition:speaker	11	5.95	<0.001
Vowel:speaker	18	127.92	<0.001
Condition:vowel:speaker	198	3.13	<0.001
Residuals (df denominator)	1192		

**TABLE II.** Fixed effects results (app, speaker) of linear regressions comparing caller's in-app recordings with the offline iPad as the reference device. (Not shown: all vowels differed from the reference /ɔ/ (*p* < 0.05) except /ʌl/ in F1, /o/ in F2, and both F1 and F2 for /a/, indicating low-back merger; ns, not significant.)

F1	Estimate	SE	df	*t*	*p*
(Intercept: iPad, female)	721.78	9.80	60.95	73.65	<0.001
App: Skype	3.44	4.37	474.91	0.79	ns
App: Teams	6.23	4.37	474.91	1.42	ns
App: Zoom	15.17	4.36	475.84	3.48	<0.001
Speaker: male	−54.89	3.09	475.28	−17.76	<0.001
F2	Estimate	SE	df	*t*	*p*
(Intercept: iPad, female)	1141.86	30.21	68.36	37.79	<0.001
App: Skype	4.37	16.20	475.83	0.27	ns
App: Teams	−7.26	16.20	475.83	−0.45	ns
App: Zoom	−14.21	16.14	476.71	−0.88	ns
Speaker: male	−186.01	11.45	476.11	−16.25	<0.001

**TABLE III.** Fixed effects results (receiver, speaker) of linear regressions comparing live recipient recordings with the caller's Zoom recording as the reference condition. (Not shown: all vowels differed from the reference /ɔ/ (*p* < 0.05) except /ʌ/ in F1, /o/ in F2, and both F1 and F2 for /a/, indicating low-back merger; ns, not significant.)

F1	Estimate	SE	df	*t*	*p*
(Intercept: Zoom caller, female)	737.22	9.88	57.50	74.64	<0.001
Recipient: NL	−1.62	3.65	342.35	−0.44	ns
Recipient: NB	2.44	3.65	342.35	0.67	ns
Speaker: male	−62.09	2.98	342.23	−20.81	<0.001
F2	Estimate	SE	df	*t*	*p*
(Intercept: Zoom caller, female)	1129.78	31.95	392.00	35.37	<0.001
Recipient: NL	−3.17	15.35	392.00	−0.21	ns
Recipient: NB	20.15	15.35	392.00	1.31	ns
Speaker: male	−165.09	12.54	392.00	−13.17	<0.001

**TABLE IV.** Fixed effects results (app, speaker) of linear regressions comparing the recipient's in-app recordings over a medium-strength internet connection with the transmitted H4n recording as the reference condition. (Not shown: all vowels differed from the reference /ɔ/ (*p* < 0.05) except F2 for /a, ul/; ns, not significant.)

F1	Estimate	SE	df	*t*	*p*
(Intercept: H4n, female)	787.05	10.61	53.96	74.15	<0.001
App: Skype	−4.99	3.06	476.79	−1.63	ns
App: Teams	−10.28	3.05	476.68	−3.37	<0.001
App: Zoom	−1.81	3.06	476.79	−0.59	ns
Speaker: male	−82.64	2.16	476.91	−38.20	<0.001
F2	Estimate	SE	df	*t*	*p*
(Intercept: H4n, female)	1138.96	28.72	58.04	39.65	<0.001
App: Skype	−2.85	11.30	476.83	−0.25	ns
App: Teams	−20.12	11.27	476.63	−1.78	ns
App: Zoom	−2.37	11.30	476.83	−0.21	ns
Speaker: male	−177.16	7.99	477.04	−22.17	<0.001

**TABLE V.** Fixed effects results (app, speaker) of linear regressions comparing the recipient's in-app recordings over a weak internet connection with the transmitted H4n recording as the reference condition. (Not shown: all vowels differed from the reference /ɔ/ (*p* < 0.05) except /ʌl/ in F2 and both F1 and F2 for /a/, indicating low-back merger; ns, not significant.)

F1	Estimate	SE	df	*t*	*p*
(Intercept: H4n, female)	814.26	11.70	54.10	69.62	<0.001
App: Skype	1.18	3.42	340.66	0.35	ns
App: Zoom	−0.31	3.43	340.81	−0.09	ns
Speaker: male	−82.10	2.80	340.76	−29.34	<0.001
F2	Estimate	SE	df	*t*	*p*
(Intercept: H4n, female)	1161.93	29.23	59.95	39.75	<0.001
App: Skype	−2.28	12.49	340.75	−0.18	ns
App: Zoom	−6.89	12.52	341.05	−0.55	ns
Speaker: male	−180.98	10.21	340.95	−17.72	<0.001

CODE 1: Wireless fidelity logging script for OS X

#log the Wi-Fi settings for every other second of transmission

#control-c will terminate

while true; do

/System/Library/PrivateFrameworks/Apple80211.framework/Versions/Current/Resources/airport -I; sleep 1; done ≫ wifitest.txt

CODE 2: OS X terminal commands to calculate SNR

#extract from log file

grep ‘agrCtlRSSI' wifitest.txt

grep ‘agrCtlNoise' wifitest.txt

Quality: $((rssi - noise)).
